# 

**Published:** 1888-10

**Authors:** 


### Society Notices.

### Central Illinois Dental Society.—Ohio State Dental Society, Reorganized.

The Fifth, Sixth, Seventh, and Eighth District Dental Societies of the State of New York will unite in a joint convention at the Leland Hotel, Syracuse, Oct. 24th, 25th, and 26th. The meeting will be called to order at 2 o'clock.

A cordial invitation is extended to the profession to be present. An extensive programme has been arranged.

Prominent dentists from Chicago, Baltimore, Cincinnati, Philadelphia, New York, Boston, Toronto, Albany, Newark, and other cities have consented to take part.

One half day will be devoted to clinics and demonstrations of improved methods of work and new appliances. All the leading dental manufacturing companies have arranged to be present with a full line of their goods. They expect to outdo previous exhibitions in this section of the State.

Among the social features of the convention will be a banquet for the dentists and a reception for the ladies.

It is expected that dentists will bring their wives.

A Ladies' Committee has been appointed.

J. L. Curtis, Chair. Bus. Com., Syracuse.

---

The Seventh annual meeting of the Central Illinois Dental Society will be held at Lincoln, Tuesday, Wednesday, and Thursday, October 9th, 10th, and 11th, 1888. Extensive preparation has been made for an interesting meeting. The following programme for papers, discussions and clinics has been prepared. T[illegible] certainly is an inducement for a large attendance. Railroads and hotels make favorable rates.

PROGRAMME.

1. Filling Root Canals (using Dunn's Syringe), Dr. J. Austin Dunn, Chicago.
2. Seamless Gold Crown............Dr. E. B. Call, Peoria.
3. Amalgam Filling...............Dr. C. P. Pruyn, Chicago.

4. Copper Amalgam Filling.....Dr. C. R. Taylor, Streator.
5. Platinum and Gold Filling,
Dr. J. A. W. Davis, Galesburg.

Thursday—Morning Session, 8:30 O'Clock.

1. Gold Filling..............Dr. J. W. Collins, Lincoln.
2. Crown—Porcelain Front...Dr. K. B. Davis, Springfield.
3. Gold Filling..............Dr. M. H. Patton, Springfield.
4. Gold Filling.................Dr. T. A. Davis, Winchester.

ESSAYS AND DISCUSSIONS

Dental Hygiene....................Dr. H. H. Fitch, Pekin.
Management of Children in the Dental Office,
Dr. Garrett Newkirk, Chicago.
Amalgams..........................Dr. C. R. Taylor, Streator.
Clinical Lecture on Amalgams...Dr. C. P. Pruyn, Chicago.
Remedies...............................Dr. E. K. Blair, Waverly.
Prosthetic Dentistry...............Dr. E. D. Swain, Chicago.
Question Drawer, conducted by Dr. J. D. Moody, Mendota.

---

The Fifth annual meeting of the Ohio State Dental Society (reorganized) will be held in Cincinnati, Oct. 16th, 17th, and 18th, 1888, in Lincoln Club Hall, Garfield Place and Race sts., session commencing at 10 o'clock a. m., Oct. 16th.

This meeting of the Ohio State Dental Society bids fair to be one of the largest in the history of the society, first on account of papers and clinics offered and second, the unsurpassed attractions of the Queen City as a metropolis. The Centennial Exposition of the Ohio Valley and Central States has never been excelled by any similar exposition in the United States and is alone an inducement for dentists and their families to visit Cincinnati. The Art Museum, Zoological Gardens and other places of interest afford additional entertainment and instruction to visitors. Rail-road rates are down to the minimum, enabling several to travel for the usual price of one. Among the hotels may be mentioned the Burnet House, St. Clair, Dennison, Palace, Hotel Emery, Grand, Gibson, St. James, Geneva. There are a number of first-class boarding houses on Garfield Place in the immediate vicinity of the Lincoln Club, where favorable rates may be had.

Any information will be cheerfully imparted by the local member of the Executive Committee, Dr. E. G. Betty, 82 Garfield Place.

PROGRAMME.

I. The Ottolengni Method of Anæsthetizing Sensitive Dentine, Dr. Levitt E. Custer, Dayton.

II. Pericementitis, Dr. F. S. Maxwell, Steubenville.

III. Machinery as Used in Dentistry, Dr. C. R. Butler, Cleveland.

IV. Implantation, Replantation, and Transplantation, Dr. H. A. Smith, Cincinnati.

V. Examination of the Teeth and Jaws in Skulls, Dr. E. G. Betty, Cincinnati.

VI. Dental Education, Dr. J. Taft, Cincinnati.

VII. Medical Education for Dentists, Dr. C. M. Wright, Cincinnati.

VIII. Voluntary Papers.

IX. Question Drawer, opened by J. Taft.

CLINICS.

Methods of Cleaning Teeth, Dr. Wm. Taft.

Operative Dentistry, Dr. D. W. Clancy.

Implantation, Dr. M. H. Fletcher.

Articulating artificial teeth by the Bomville Method, Dr. Grant Molyneaux.

Voluntary Clinics.

Exhibition of Appliances.

**A MONTHLY MAGAZINE DEVOTED TO THE INTERESTS OF THE**

### DENTAL PROFESSION.

*Terms Reduced to* $2.00 *Per Year. Single Copies,* 25 *Cents.*

### EDITED BY PROF. J. TAFT, D.D.S.

—AND—

### Published by SAM'L A. CROCKER & CO., Ohio Dental and Surgical Depot.

The volume commences in January and closes in December.

The REGISTER being issued monthly is always fresh, therefore Indispensable to the practitioner who desires to keep posted up with the new inventions and improvements which are daily developed. Neither expense nor effort will be spared to give value and variety to its contents. Articles will be illustrated with well executed engravings whenever they will add to the interest or assist to explanation.

Its extensive circulation and frequency of issue make it one of the BEST DENTAL ADVERTISING MEDIUMS in the United States.

All subscriptions must begin with January or July.

Local or special notices, five lines or less, will be inserted for $3 each insertion; over five lines, 50 cts. per line.

All advertising bills payable in advance, or at furthest, after the issue of the first number containing the advertisement. All transient advertisements to be paid for in advance.

☞CONTRIBUTIONS SOLICITED.

Exclusively Editorial Communications should be addressed to the Editor.

The latest number of the REGISTER may be ordered with or without other goods at any time, and all letters should be addressed to

**SAM'L A. CROCKER & CO., Ohio Dental and Surgical Depot, Cincinnati, O.**

